# Improving hospitalization in children and adolescents through animal assisted interventions (AAIS): A systematic review

**DOI:** 10.1192/j.eurpsy.2021.1242

**Published:** 2021-08-13

**Authors:** C. Correale, M. Borgi, B. Collacchi, T. Grimaldi Capitello, F. Vigevano, F. Cirulli, S. Cappelletti

**Affiliations:** 1 Clinical Psychology, Bambino Gesù Children Hospital, Rome, Italy; 2 Center For Behavioural Sciences And Mental Health, Istituto Superiore di Sanità, er for Behavioural Sciences and Mental Health, Rome, Italy; 3 Neurological Sciences, Bambino Gesù Children Hospital, Rome, Italy

**Keywords:** humanization of care, stress and pain reduction, Animal Assisted Interventions, Pediatric Hospital

## Abstract

**Introduction:**

Animal Assisted Interventions (AAIs) are increasingly introduced in pediatric care settings as a mean to promote the physical, mental, and emotional well-being of hospitalized children and adolescents and the humanization of the hospital environment.

**Objectives:**

The aim of this work was to review published studies implementing AAIs in hospital settings and to assess their effectiveness in reducing stress and pain, ameliorating social behavior, quality of life, and mood in pediatric patients. Reviewed interventions were also evaluated for their effects on caregiver’s stress and burden, as well as on perception of the work environment in hospital staff.

**Methods:**

Studies were systematically searched using PubMed, Scopus, ProQuest and Web of Science databases in accordance with PRISMA guidelines. The search was aimed at identifying studies examining the effects of AAIs on behavioral and physiological response to stress in children and adolescents (0-18 years) formally admitted to a hospital for a stay, as well as in those undergoing a visit for treatments or medical examinations.

**Results:**

Of 350 studies screened, 17 were eligible for inclusion. Most of them focused on stress, pain and anxiety reduction in pediatric patients, and used both physiological parameters and behavioral observations/scales. The vast majority of the studies employed dogs. Results show the potential of AAIs to reduce anxiety and behavioral distress in pediatric patients, while acting on physiological measures associated with arousal.

**Conclusions:**

Although further studies of better quality are still needed, the findings of this review may have implications for clinical practices suggesting appropriate planning of AAIs by pediatric healthcare professionals.

